# Current and future perspectives for biomass waste management and utilization

**DOI:** 10.1038/s41598-024-59623-1

**Published:** 2024-04-26

**Authors:** Ornella Francioso

**Affiliations:** Department of Agricultural and Food Sciences, Alma Mater Studiorum, University of Bologna, Viale G. Fanin 40, 40127 Bologna, Italy

**Keywords:** Geochemistry, Biotic

## Abstract

The collection is dedicated to the conversion of biomass wastes into value-added bioproducts and bioactive compounds, with a focus on their applications in the agro-energy sector. Of particular relevance were three studies evaluating the effects of livestock waste, rice straw and biochar on soil properties, crops and productivity. Of note were two articles on the bioconversion of aquaculture sludge by insects and the production of biofuel from seed oil as an alternative to overcoming the depletion of fossil fuels. Finally, one article analysed the potential for recovering organic and mineral compounds from gastropod shells. The articles provided insights into the management and use of biomass wastes, as well as suggestions for future research to promote sustainability in agriculture.

Due to the COVID-19 pandemic and ongoing conflicts, there are rapid and unprecedented changes occurring in the climate, environment, and economy. Therefore, it is necessary to find immediate solutions to these challenges. The goal is to identify and co-develop innovative circular solutions that promote environmental sustainability, resilience, and economic growth in the near future. Agriculture has traditionally operated as a circular economic system, utilizing livestock farming yields waste as manure, animal bedding, and related by-products and crop residue^1^ to restore soil fertility. This has been achieved through the use of elemental agricultural practices, taking into account the availability of biomass waste. Advances in knowledge, technology, and synthetic products have significantly increased primary productivity, food production processes, and food security. As a result, the circularity has been lost or reduced, resulting in a return to a linear economic model with a considerable accumulation of waste^2^. Mismanagement of biomass waste has significant environmental impacts due to the GHG emission, pollutants, and unpleasant odours. According to Gerber et al.^3^, agricultural and livestock by-products are responsible for 40% of global CH_4_ emissions, while waste disposal accounts for 18%^3^. Therefore, managing biomass waste properly is crucial in mitigating its impact on the climate and environment^4^. The 4Rs, Reduce, Reuse, Recover, and Recycle^5^ became mandatory. By using biomass waste, local communities can increase their income, reduce the financial burden of waste disposal and minimize their carbon footprint^6^.

## Article highlights in this collection

This Collection focuses on the conversion of biomass waste into value-added bio-products and bioactive compounds, and their applications in the agro-energy sector. The ultimate aim is to reduce waste and promote a sustainable and environmentally friendly approach. Three studies analyzed the effects of livestock waste, rice straw^7,9^ and biochar^10^ on various soil characteristics, crops and productivity. Two papers are presented focusing on how insects bio-convert aquaculture sludge into nutrients^11^, and finding alternative energy sources to overcome the fragility of fossil fuels and their negative environmental effects^12^. Finally, in one paper, the potential for recovering organic and mineral compounds from gastropod shells was explored^13^.

Specifically, Wang et al.^7^ investigated the recycling of P from livestock waste, including poultry manure (PM), cattle manure (CM), cattle bone meal (CB), maize straw crop residues (MS), and superphosphate (SSP), in acidic (red soil) and alkaline (river soil) soils that received the same total P supply. The study addressed the problem of low P availability in these soils, which is a major constraint to sustainable crop productivity. Only CM increased soil labile P fractions to levels similar to SSP. These results have important implications for sustainable agriculture, as phosphate fertilizers are produced from phosphate rock, a non-renewable resource^8^.

Ghosh et al.^9^ used rice straw at 12 Mg ha^−1^ combined with P-solubilizing microorganisms and 75% P mineral to improve silicon (Si), organic acid levels, soil enzyme activity and grain yield. The results showed that grain yield increased by 40% compared to no P application, and by 18% compared to 100% P application. Therefore, recycling agricultural residues with phosphorous-solubilizing microorganisms has the potential to increase crop yields and save 25% of mineral P, leading to sustainable soil management.

Pinnavaia et al*.*^10^ addressed the impact of biochar in agriculture. They evaluated the effects of sugarcane bagasse biochar on the growth of cotton (*Gossypium hirsutum* L.), as well as the yield and quality of the lint, in a three-year field experiment. Despite the high concentration of biochar added to the soil, no significant effect on cotton lint and seed yield was observed in the first two years. The agricultural and environmental implications of this research are significant, as it highlights the potential of biochar to promote sustainability in agriculture.

An interesting way to promote the transition to a circular economy is to use insects to bio-convert aquaculture sludge to recover nutrients that can be used as animal feed. In addition, the residual material can be conveniently used as organic fertilizer. Rossi et al.^11^ used black fly larvae (*Hermetia illucens*) to treat aquaculture sludge waste at different amounts. Overall, this study highlights the potential of *Hermetia illucens* larvae in managing fresh aquaculture sludge waste, providing a dual benefit of waste reduction and insect biomass production.

Jan et al.^12^ studied a new method for producing biofuel from *Sisymbrium Irio* L. seed oil using a titania catalyst. The authors highlight the significance of this plant as it can be grown in unfavourable environments and, due to its physicochemical properties, produces an eco-friendly fuel that is a competitive source for commercial biodiesel production.

Elegbede et al.^13^ investigated the composition of gastropod shells (*Tympanotonus fuscatus*, *Pachymelania aurita*, and *Thais coronata*) as a way to recover CaCO_3_ and other organic molecules from readily available biomass waste. The use of these materials has proven to be essential and cost-effective in promoting a circular bio-economy.

The articles presented valuable insights into biomass waste management and utilization, as well as suggestions for future research to promote sustainability in agriculture.
